# A Case Report on Metamizole-Induced Agranulocytosis: Is the Benefit Worth the Risk?

**DOI:** 10.7759/cureus.34467

**Published:** 2023-01-31

**Authors:** Rita Carvalho, Célia Henriques, Marco Fernandes, Cláudio Gouveia, Catarina Gama

**Affiliations:** 1 Internal Medicine, Hospital de São Francisco Xavier, Lisbon, PRT

**Keywords:** fever, neutropenia, agranulocytosis, dipyrone, metamizole

## Abstract

Metamizole is a drug with analgesic and antipyretic properties widely available in Portugal. Its use is highly controversial because of the risk of agranulocytosis, a rare but serious adverse event. A 70-year-old female patient with a recent history of treatment with metamizole for post-surgery fever and pain presented to the ED with sustained fever, diarrhea, and painful mouth ulcers. Laboratory tests revealed agranulocytosis. The patient was placed under protective isolation and started treatment with granulocyte-colony stimulating factor (G-CSF) and empiric antibiotic therapy with piperacillin/tazobactam and vancomycin for neutropenic fever. After an extensive workup, no source of infection was identified. During hospitalization, infectious and neoplastic causes of agranulocytosis were investigated, but the results were negative. Metamizole-induced agranulocytosis was suspected. The patient completed a total of three days of G-CSF and eight days of empiric antibiotic therapy with sustained clinical improvement. She was discharged completely asymptomatic and remained clinically stable during follow-up without a resurgence of agranulocytosis. This case report is intended to increase awareness of metamizole-induced agranulocytosis. While this is a well-known side effect, it is also often overlooked. It is paramount that both physicians and patients know how to correctly manage metamizole to prevent and promptly treat agranulocytosis.

## Introduction

Metamizole (dipyrone) is a pyrazolone derivative with analgesic, antipyretic and spasmolytic properties that has been available since 1922. Scientific evidence supports its effectiveness in treating severe acute and chronic pain and for fever that does not respond to other treatments. This substance is available in several European Union (EU) member states. However, there has been a longstanding controversial debate regarding its safety, particularly the risk of agranulocytosis [1-3]. Agranulocytosis is generally defined as a decrease in the peripheral neutrophil count to less than 500 cells/µL. This reaction might lead to life-threatening and even fatal concurrent infections. For this reason, metamizole was never approved or has been withdrawn from the market in many other countries like the United States of America (USA), Canada, Australia, the United Kingdom (UK), France, Norway, and Sweden [3,4]. In a retrospective analysis of the EudraVigilance database, which collects information on suspected adverse reactions to medicines authorized in the European Economic Area (EEA), from 1985 to 2017, there were 1448 spontaneous reports of suspected metamizole-associated agranulocytosis from 31 different countries [5]. Most cases were reported by Germany, Spain, and Switzerland. Furthermore, according to the Portuguese National Authority for Medications and Health (INFARMED), between 2008 and 2018, there were 11 reported cases of agranulocytosis potentially associated with metamizole [6]. This means a frequency of one to two cases per year, which is within the expected frequency of a very rare reaction. However, this is a minimal risk since underreporting is known to be substantial for pharmacovigilance data. This case report describes an episode of agranulocytosis attributed to metamizole.

## Case presentation

A 70-year-old female patient with arterial hypertension, dyslipidemia, vertigo, and a history of C4-C5 herniated disc surgery underwent an elective anterior cervical discectomy and fusion for a C5-C6 herniated disc. Her medication included amlodipine, valsartan, atorvastatin, and betahistine. Laboratory tests before surgery were unremarkable. There were no acute complications, and she was discharged from the hospital with paracetamol and metamizole as needed for pain and/or fever. On the day of discharge, the patient presented with a fever with no other associated symptoms, and she started taking paracetamol and metamizole as prescribed. Initially, the fever responded to the antipyretic treatment but later reappeared and became increasingly frequent. Three weeks after surgery, she was admitted to the ED for persistent fever associated with the recent development of diarrhea and painful mouth ulcers. She denied respiratory, cardiovascular, neurological, or genital symptoms. At the time of admission, the patient was febrile (tympanic temperature 38.9ºC), slightly hypotensive (blood pressure 105/44 mmHg), and tachycardic (heart rate 110 bpm). On examination, she had a hyperemic oropharynx and two aphthous ulcers. The anterior cervical scar from the recent surgery showed no signs of infection. Pulmonary auscultation and abdominal examination were unremarkable. There was no skin rash or palpable lymph nodes. The thoracic radiograph was normal. Laboratory tests revealed agranulocytosis and elevation of C-reactive protein (Table 1).

**Table 1:** Laboratory findings at admission, discharge, and follow-up.

Laboratory parameters	Admission	Discharge	Follow-up	Normal range
Hemoglobin (g/dL)	10.6	10.9	11.5	12.0-15.0
Leukocytes (x10^9/L)	1.1	21.5	8.4	4.0-10.0
Neutrophils (x10^9/L / %)	0.00/0.4	17.31/80.5	5.29/63.0	40.0-80.0%
Lymphocytes (x10^9/L / %)	0.97/87.9	2.73/12.7	2.18/26.0	20.0-40.0%
Monocytes (x10^9/L / %)	0.13/11.4	1.40/6.5	0.67/8.0	2.0-11.7%
Eosinophils (x10^9/L / %)	0.00/0.3	0.00/0.0	0.17/2.0	1.0-6.0%
Basophils (x10^9/L / %)	0.00/0.0	0.06/0.3	0.08/1.0	0.0-2.0%
Platelets (x10^9/L)	419	328	465	150-400
C-reactive protein (mg/dL)	17.6	2.21	0.46	<0.5

The SARS-CoV2 test was negative. Cervical and thoracic spinal CT revealed C4-C7 surgical intervention with no signs of infection. A diagnosis of neutropenic fever was confirmed, and the patient was placed under protective isolation. Blood and urine cultures were collected. Since there was a concern about whether the patient was evolving to septic shock, treatment with G-CSF and empiric antibiotic therapy with piperacillin/tazobactam and vancomycin was started.

During the investigation, blood and urine cultures as well as an extensive viral screen panel, which included serology for Epstein-Barr Virus (EBV), Cytomegalovirus (CMV), Hepatitis B Virus (HBV), Hepatitis C Virus (HCV), and HIV, were negative for acute infection. Clostridium difficile toxins on the stool were negative. Protein electrophoresis showed hypergammaglobulinemia compatible with chronic infection/inflammation. Folate and vitamin B12 levels were normal. Peripheral blood smear confirmed neutropenia and revealed rarely stimulated lymphocytes with no blasts or other immature cells. Abdominal sonography and CT revealed a slight hepatic enlargement with a normal spleen and a simple left renal cyst without any suspicious traits. Clinical history was reviewed in detail, with special attention being given to recent medication taken by the patient, and metamizole-induced agranulocytosis was suspected.

During hospitalization, the patient remained hemodynamically stable and afebrile. There was no need for volume resuscitation or vasopressors. Diarrhea lasted a total of four days, and the aphthous stomatitis started healing. A progressive increase in granulocyte count and a decrease in C-reactive protein levels were noted. After completing three days of G-CSF and eight days of empiric antibiotic therapy, the patient was discharged entirely asymptomatic. The hemogram at discharge revealed a rebound leukocytosis and neutrophilia (Table 1). She was advised to suspend metamizole and return to the hospital for reevaluation.

At reevaluation, two weeks later, the patient remained afebrile and asymptomatic with a normal hemogram and inflammatory markers (Table 1).

## Discussion

In the presented report, we describe a case of presumed metamizole-induced agranulocytosis. The patient had been taking metamizole for postoperative pain and fever, which is within the approved indications for the drug in Portugal [6]. Agranulocytosis was confirmed three weeks later by peripheral blood counts. During the investigation, no alternative causes, such as malignant disorders or viral infections, were identified. The patient’s medical history and use of concomitant drugs were non-significant for the development of agranulocytosis. This case was reported and analyzed by INFARMED, and the causality between the drug and the adverse reaction was classified as probable. A strategy to better understand the risk of developing agranulocytosis using metamizole would be to compare the number of prescriptions issued and the number of patients with the adverse reaction. However, to our knowledge, there are no articles in the Portuguese literature regarding this subject. The Berlin Case-Control Surveillance Study calculated an incidence rate of 0.96 cases per million per year [2]. In comparison, data from the Metropolitan Area of Barcelona estimated a slightly lower incidence rate of 0.56 cases per million per year [7]. An analysis of reports from Switzerland estimated a minimal incidence rate of 0.46-1.63 cases per million person-days of use [1]. Estimates of agranulocytosis incidence vary widely in the literature because most studies use pharmacovigilance systems data which, mainly due to underreporting, do not yield reliable information about the frequency of adverse drug reactions [8]. In a matched control German cohort, the risk for developing drug-induced agranulocytosis and neutropenia after metamizole prescription was 1:1602 [8]. A Swedish investigation had similar results, with an estimated incidence of 1:1439 [9].

A relevant topic worth discussing is whether this particular patient had any important risk factors for developing this adverse reaction. In the retrospective analysis of the EudraVigilance database, the risk of agranulocytosis was higher with concomitant use of other drugs (especially methotrexate), allergies or drug hypersensitivity, autoimmune diseases or hepatitis [5]. Another retrospective study identified several possible risk factors, namely a history of allergy, previous leucopenic episodes, HCV infection, and concomitant use of cytostatic drugs [4]. On the other hand, a German study that analyzed 161 reports of metamizole-induced agranulocytosis failed to identify specific factors that enhance the risk of this adverse reaction [3]. Even though this patient did not have any history of allergies, previous adverse drug reactions, diseases, or relevant medication, identifying patients with these risk factors might allow targeted and safer metamizole use. According to the patient, she took metamizole intermittently with an average daily dose within the recommended range (one pill of 575 mg every eight hours). Even though there is inherent uncertainty regarding dosing and duration of therapy in individual cases, data from several case reports agree that metamizole-induced agranulocytosis is not a result of dose-dependent toxicity but rather an idiosyncratic reaction associated with immunological and metabolic susceptibility factors [1,2,4,7]. Considering the duration of therapy, in this case, the patient developed agranulocytosis in less than three weeks and denied having ever taken metamizole before. This latency time is coherent with most reports. In the EudraVigilance study, the median time between starting metamizole and developing agranulocytosis was 13 days and much shorter in patients who had already received metamizole before [5]. These findings are also consistent with Swiss investigations, where the median latency time was between seven and 14 days [1,4]. Analyses from Sweden, Germany, and Spain had similar results, with most cases occurring within six weeks of permanent or intermittent metamizole treatment [3,7,9]. Ibáñez L et al. suggest that the risk of agranulocytosis increases with the duration of use and that it disappears 10 days after the last dose of metamizole [7]. Although metamizole-induced agranulocytosis is not a dose-dependent reaction, the authors consider that higher doses or longer exposure periods are more likely to induce sensitization. Once a patient has become sensitized to the drug, the severity of the reaction is probably unrelated to the dose taken. Agranulocytosis is a serious adverse drug reaction, and it might become life-threatening. In the EudraVigilance investigation, about 16% of cases ended fatally [5]. Some studies noted a decrease in mortality rate over time, probably associated with increased alertness among doctors, prompt discontinuation of the offending drug, and treatment with effective broad-spectrum antibiotics and/or G-CSF [9]. Other studies, however, reported an increase in fatal cases in parallel with the increased reporting rate and use of metamizole despite the option for G-CSF treatment [1]. In several analyses, old age, sepsis or pneumonia, concomitant medication with methotrexate, and pancytopenia were associated with an increased risk of severe complications and fatal outcomes [1,3].

While metamizole-induced agranulocytosis is a well-known side effect, there are no established management protocols to minimize this risk. First, it is imperative to identify strong risk factors for developing agranulocytosis under metamizole. These could be used to determine patients for whom metamizole should be avoided. Current studies are mainly based on case reports of pharmacovigilance systems. Although spontaneous reporting is an important and cost-efficient way of detecting infrequent adverse drug reactions, assumptions about utilization patterns may not always correctly reflect the actual use leading to biased risk estimations [9,10]. In order to examine a rare but severe adverse reaction, long-term controlled trials with large populations are essential [8]. Considering that as the number of prescriptions increases, the number of reports also increases accordingly, another evident way to reduce the risk of agranulocytosis is to reduce the number of patients under treatment with metamizole [3]. This goal could be achieved by strongly discouraging off-label use through notifications about the indications and risks of metamizole. The risk of agranulocytosis should be considered in comparison with other life-threatening adverse effects of analgesics and anti-inflammatory medication (for example, GI bleeding) [7]. INFARMED’s last notification to healthcare professionals was in 2018. They recommended not using metamizole in patients with a history of blood dyscrasias and treatment with immunosuppressants or other medication that may cause agranulocytosis [6]. They also advise particular attention when prescribing it to older patients. Lastly, they consider that metamizole maintains a positive risk/benefit ratio as long as all indications and precautions are met. After starting treatment with metamizole, it is essential to inform patients about the risk and advise them to seek immediate medical attention whenever fever, sore throat, or other signs of infection occur [4,8]. Healthcare professionals must be notified that early diagnosis with WBC count and withdrawal of metamizole is mandatory in these cases [1,11]. Patients should be monitored and treated according to the severity of their symptoms [11]. INFARMED recommends that metamizole be used for a restricted time of seven days; if longer, blood cell counts must be monitored [6]. However, agranulocytosis can emerge at highly variable intervals ranging from the first day to months after treatment with metamizole has begun [11]. Routine blood count monitoring during metamizole therapy needs to be evaluated in prospective randomized trials in order to determine time intervals and the benefits and costs of a screening program [4]. Currently, there is no conclusive evidence-based recommendation for routine monitoring of blood cell counts. Therefore, the onset of clinical symptoms should be used as a trigger for this analysis [11].

## Conclusions

Agranulocytosis is a well-known potential adverse reaction of metamizole. Although frequently classified as very rare, it is an underreported situation with severe and potentially life-threatening outcomes. This case demonstrates the need for a thorough clinical history of past medical illnesses and concomitant medication before prescribing this drug. Prospective randomized trials with large populations are fundamental to identifying patients at greater risk for agranulocytosis and establishing a follow-up plan.
